# In Situ Formation of WC/W_2_C Heterostructures on N-Doped Carbon for Deep Oxidative Desulfurization of Fuel Oil

**DOI:** 10.3390/molecules30030617

**Published:** 2025-01-31

**Authors:** Peng Zuo, Fuyan Zhao, Fanfan Liu, Jinpei Hei, Guozheng Lv, Xianzong Huang, Jun Zhang, Meng Zhang, Yefeng Liu, Tao Ma

**Affiliations:** Engineering Technology Research Center of Preparation and Application of Industrial Ceramics of Anhui Province, Engineering Research Center of High-frequency Soft Magnetic Materials and Ceramic Powder Materials of Anhui Province, School of Chemistry and Material Engineering, Chaohu University, Chaohu 238000, China; zp@chu.edu.cn (P.Z.); fyzhao@chu.edu.cn (F.Z.); lff@chu.edu.cn (F.L.); 053074@chu.edu.cn (J.H.); 14755796996@163.com (G.L.); 15156256409@163.com (X.H.); 13339143725@163.com (J.Z.); 15357423324@163.com (M.Z.)

**Keywords:** oxidation desulfurization, polyaniline, tungsten carbide heterojunction, electron transfer, N-doped carbon

## Abstract

A novel tungsten-based heterojunction nanocomposite material was developed for the efficient oxidative desulfurization (ODS) of fuel oil, enabling the production of low-sulfur fuel and a reduction in harmful SO_x_ emissions. In this material, the WC/W_2_C heterojunction was uniformly immobilized on a porous nitrogen-doped carbon (NC) matrix structure through facile in situ pyrolysis of polyaniline–phosphotungstic acid (PANI/PTA) precursors. The resultant WC/W_2_C@NC catalyst demonstrated remarkable desulfurization performance, achieving 100% removal of 4000 ppm dibenzothiophene (DBT) in just 15 min at 60 °C in the presence of 0.03 g of WC/W_2_C@NC and a H_2_O_2_/S molar ratio of 2. This exceptional activity is attributed to the synergistic effects stemming from the accelerated electron transfer by the NC matrix, the intricate porous network, and the abundant WC/W_2_C heterojunction active sites. Moreover, the in situ formation of NC around WC/W_2_C mitigated active site leaching, ensuring remarkable stability, with a DBT removal rate of 97.2% maintained even after eight recycling cycles. This work provides a versatile and scalable approach for fabricating tungsten-based heterojunction catalysts and highlights the potential of WC/W_2_C@NC as a high-performance, durable ODS catalyst, paving the way for further advancements in sustainable desulfurization technologies.

## 1. Introduction

Despite the ongoing growth of the global economy and significant advancements in the renewable energy sector, petroleum remains a crucial component of the current energy landscape, contributing to over 30% of the global energy mix. Nevertheless, the excessive extraction and consumption of petroleum resources present a major challenge, particularly as the quality of crude oil deteriorates annually due to rising sulfur content. The sulfur oxides emitted during the combustion of high-sulfur crude oil not only pollute the atmosphere and pose health risks but also damage industrial catalytic converters, especially those in vehicle exhaust gas purification systems, thereby reducing the purification efficiency [1,2]. Addressing these issues necessitates controlling the production of SO_x_ pollutants at their source, with advanced desulfurization technology as the pivotal solution.

The primary desulfurization technology employed in industry is hydrodesulfurization (HDS), which transforms the sulfur in oil products into H_2_S gas, effectively reducing the sulfur content [3]. Although HDS efficiently removes small-molecule sulfides, the catalytic elimination of thiophene sulfides such as dibenzothiophene (DBT), 4,6-dimethyldibenzothiophene (4,6-DMDBT), and benzothiophene (BT) necessitates high temperatures, high pressures, and substantial hydrogen consumption, which limits its removal effectiveness [4]. Consequently, there is growing interest in alternative non-hydrodesulfurization technologies, including oxidative desulfurization (ODS) [5,6], adsorptive desulfurization [7,8,9], extractive desulfurization [10,11], photocatalytic desulfurization [12,13], and biodesulfurization [14,15]. Among these, ODS stands out as effectively complementing HDS due to its mild reaction conditions, lower equipment costs, and strong ability to remove thiophene [16]. ODS oxidizes stubborn sulfur compounds into highly polar sulfoxides and/or sulfones, which can be readily separated and recovered through methods such as adsorption, extraction, distillation, and crystallization [17,18].

Highly active catalysts are crucial for the efficiency of ODS. To date, various transition-metal oxides (e.g., WO_3_, MoO_3_, and MoO_x_) [19,20,21], nitrides (e.g., W_x_N) [22], and carbides (including M_x_C, where M = W or Mo) [23,24] have been explored as potential ODS catalysts, all showing considerable developmental promise. Specifically, our previous research indicates that transition-metal carbide catalysts, including W_2_C [24] and Mo_2_C [23], hold considerable potential for ODS catalysis [25,26]. For instance, Wang et al. [24] reported the synthesis of N-doped carbon-coated tungsten carbide (WₓC@NC) via the one-step annealing of a precursor formed by combining metal phthalocyanines (MQPcs, M = Zn, Co, Fe, Cu, Ni, Mn) with Keggin- and Dawson-type phosphotungstic acids (POMs). The resulting W_x_C@NC demonstrated excellent ODS performance, achieving complete oxidation and the removal of 500 ppm of DBT within 40 min, and it revealed the influence of different metal types on carbide formation during annealing. Despite their potential, carbide catalysts face challenges in ODS systems, such as the limited mass transfer of sulfides at low concentrations and complex post-treatment processes for oxidation products. To enhance the activity of transition-metal carbide catalysts in ODS, immobilizing them on particular supports with unique structural and functional properties has become a key research direction. These supports include zeolites [27], Al_2_O_3_ [28], SiO_2_ [29], and nanoporous/hybrid carbons [30,31], enabling the functionalization of catalysts and precise tuning of active sites.

Carbonaceous materials are widely utilized as functional carriers due to their affordability and tunable physicochemical and electronic properties. In particular, doping the carbon matrix with nitrogen atoms significantly boosts the electron-donating capacity of carbon materials and enhances the activity of transition-metal catalysts [32,33]. For example, Guo et al. [20] synthesized a porous, hollow, spherical MoO_x_/C catalyst through chelating dopamine with molybdate ions, followed by pyrolysis and oxidation. This process, supported by the carbon matrix, effectively prevented the excessive agglomeration of the molybdenum oxide during pyrolysis and ensured its even distribution within the carbon framework. Zhou et al. [34] demonstrated that the in situ loading of Ov-SiW_12_ onto a nitrogen-doped carbon (NC) matrix exhibited remarkable ODS activity and reusability, surpassing those of SiW_12_/C without nitrogen doping. This highlights the beneficial impact of nitrogen doping on improving ODS activity. In addition, it is recognized that heterostructure designs can often enhance the ODS catalyst activity by facilitating charge transfer, particularly by accelerating electron transfer [35,36]. Therefore, it is promising to load carbides onto nitrogen-doped supports and apply heterojunction engineering to further optimize the electron transfer pathways for improving ODS performance.

In this work, a novel porous disordered WC/W_2_C@NC catalyst was developed by firmly anchoring WC/W_2_C heterojunctions onto nitrogen-doped carbon nanosheets (NSs). The catalyst was synthesized by high-temperature pyrolysis and carbonization under an inert atmosphere, utilizing a mixture of phosphotungstic acid (PTA) and aniline (PANI) as precursors. This synthesis approach leveraged the confinement effect of the NC matrix, which promoted a high dispersion of WC/W_2_C nanoparticles and robust integration into the carbon substrate, thereby significantly enhancing the structural stability of the composite. Furthermore, the incorporation of the WC/W_2_C heterojunctions fine-tunes the electronic structure of the tungsten sites, which is a crucial factor in increasing the catalytic activity of the catalyst. The resulting WC/W_2_C@NC catalyst demonstrated outstanding ODS performance, achieving complete desulfurization within only 15 min under mild conditions for 4000 ppm of DBT at a theoretical H_2_O_2_/S molar ratio of 2 and catalyst dosage of 0.05 g. Additionally, a series of radical scavenging experiments provided insights into the ODS mechanism of the WC/W_2_C@NC catalyst.

## 2. Results

### 2.1. Synthesis and Characterization of Catalysts

**Figure 1:**
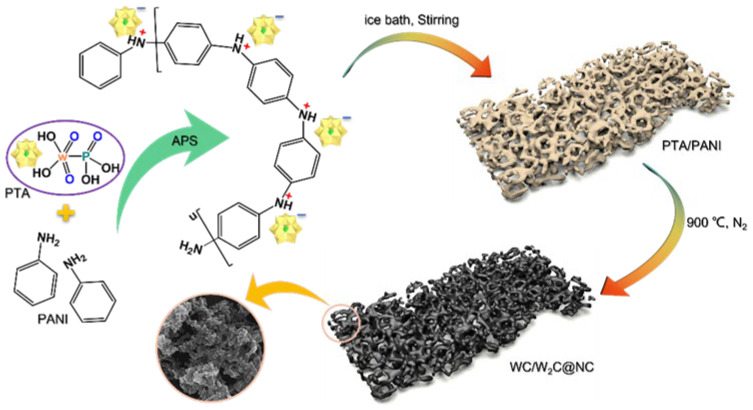
Schematic representation of the synthesis process of WC/W_2_C@NC.

The synthesis process of WC/W_2_C@NC is illustrated in Figure 1. First, PTA was used to oxidize and initiate the polymerization of aniline monomers via a simple one-step ice bath method, yielding a PTA/PANI precursor. Detailed synthesis steps can be found in Section S3 of the Supplementary Information. Subsequently, the PTA/PANI precursor was placed in a crucible and transferred into a furnace under a nitrogen atmosphere. The obtained sample was then annealed at a heating rate equal to 5 °C/min to target temperatures of 500 to 1000 °C, with a holding time of 2 h to obtain the calcined products. Based on the XRD analysis, these products were identified as PTA@NC-500, WO_3_@NC-700, W/W_2_N@NC-800, WC/W_2_C@NC-900, and WC/W_2_C@NC-1000, corresponding to annealing temperatures of 500, 700, 800, 900, and 1000 °C, respectively. Unless otherwise specified, WC/W_2_C@NC-900 is represented as WC/W_2_C@NC.

**Figure 2:**
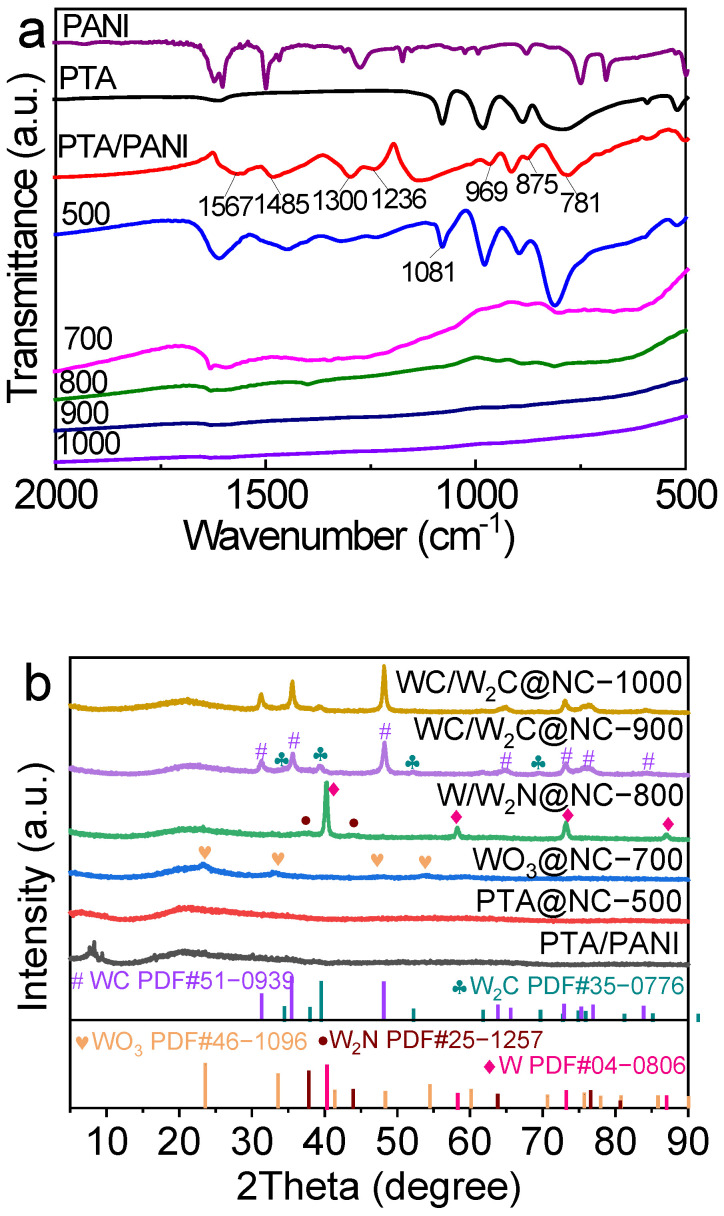
(**a**) The obtained FT-IR profiles and (**b**) XRD spectra for the PTA/PANI precursor and its annealed products at various temperatures (500–1000 °C).

Figure 2a illustrates the FT-IR spectra of the PTA, PANI, and PTA/PANI precursors, along with their annealing products at various temperatures. In the spectrum of the PTA/PANI precursor, apart from the primary characteristic absorption peaks at 1567/1485 and 1300 cm^−1^ corresponding to the C=C and C–N bonds in PANI, additional peaks at 969, 875, and 781 cm^−1^ were observed. These peaks are attributed to the stretching vibrations of the W=O_d_, W–O_b_–W, and W–O_c_–W bonds, respectively [37], indicating the successful assembly of the PTA/PANI composite. The UV-Vis spectrum of PTA/PANI (Figure S1) displayed absorption peaks at 194 and 263 nm, corresponding to the charge transfer from the terminal oxygen to tungsten (O_d_→W) and oxygen–tungsten bridges (O_b_/O_c_→W), respectively [38], further confirming the presence of PTA in the composite. Upon calcination of the PTA/PANI precursor between 500 and 1000 °C, there were significant alterations in the characteristic peaks. As the annealing temperature increased, the characteristic peaks of polyaniline and PTA gradually diminished and ultimately disappeared, likely due to the carbonization of polyaniline and the transformation of the PTA structure. The TG analysis of the PTA/PANI precursor (Figure S2) revealed significant weight loss from 140 to 1000 °C. Specifically, the weight loss below 140 °C was mainly ascribed to the desorption of physically and chemically adsorbed water on the composite material. Between 140 and 640 °C, the weight loss was mainly due to the pyrolysis of PANI, while the weight loss between 640 and 1000 °C was mainly related to the decomposition of PTA into other tungsten-containing compounds. These changes provide further evidence that the structures of PANI and PTA are significantly disrupted at high temperatures. Additionally, the post-calcination UV-Vis spectra revealed that the absorption peak of the composite at 263 nm gradually broadened with increasing temperature, indicating the progressive destruction of the PTA structure. These findings were consistent with the TG and FT-IR results.

Figure 2b displays the XRD spectra of annealed products at different temperatures. For PTA@NC-500, similarly to the PTA/PANI precursor, no distinct crystalline diffraction peaks were observed, indicating a uniform dispersion of PTA within the carriers. In the XRD pattern of WO_3_@NC-700, prominent diffraction peaks appeared at 23.64°, 33.63°, 48.43°, and 54.57°, corresponding to the (200), (220), (400), and (420) crystal planes, respectively, suggesting the transformation of PTA into WO_3_. As the temperature increased to 800 °C, peaks indicative of metallic tungsten (W) were noted at 40.1°, 58.1°, 73.0°, and 86.9°, along with peaks indicative of tungsten nitride (W_2_N) at 37.4° and 43.6°. At 900 °C, distinct diffraction peaks at 31.51° (001), 35.64° (100), 48.30° (101), 65.50° (002), 73.05° (111), 75.74° (200), and 84.15° (201), characteristic of tungsten carbide (WC) (PDF#51-0939), were observed, with additional peaks of tungsten monocarbide (W_2_C) observed at 34.28°, 39.18°, 52.12°, 61.66°, 69.51°, and 75.85°, indicating that tungsten species in WC/W_2_C@NC predominantly comprised WC and W_2_C. The XRD peaks of W_2_C sharpened upon further raising the temperature to 1000 °C, suggesting an increase in grain size at high temperatures. Based on these findings, it can be inferred that the tungsten species in the PTA/PANI precursor undergo the following transformation with increasing annealing temperature: PTA→WO_3_ (700 °C)→W/W_2_N (800 °C)→WC/W_2_C (900 and 1000 °C).

**Figure 3:**
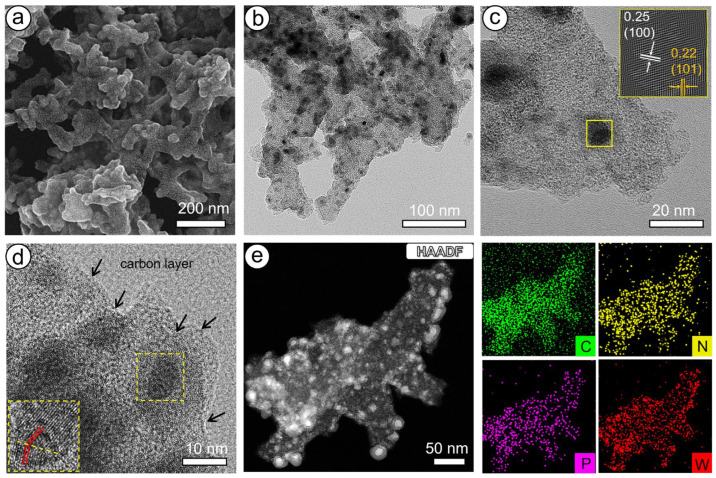
(**a**) SEM, (**b**) TEM, (**c**,**d**) HRTEM, and (**e**) HAADF images and EDX spectra of WC/W_2_C@NC. (the arrows points to the carbon layer, yellow box, the yellow box is the lattice amplification area).

The morphology and composition of the PTA/PANI precursor and WC/W_2_C@NC were analyzed using SEM, TEM/HRTEM, and elemental mapping. As shown in Figure S3a, the PTA/PANI precursor exhibits a three-dimensional porous structure composed of interwoven nanoclusters, which could provide a larger specific surface area, expose more active sites, and favor mass transfer. After annealing, the resulting WC/W_2_C@NC still retained a unique 3D porous structure (Figure 3a), indicating structural stability. Further TEM images (Figure 3b) revealed numerous black nanoparticles densely and uniformly distributed within the nitrogen-doped carbon matrix derived from the PANI polymer. HRTEM images (Figure 3c), along with reverse fast Fourier transform (FFT) images of the selected region (inset of Figure 3c) and an average of six measured lattice spacings (Figure S4a), showed lattice fringes of 0.25 and 0.22 nm on these black nanoparticles. These fringes corresponded to the (100) crystal plane of WC and the (101) crystal plane of W_2_C, respectively, confirming the formation of WC/W_2_C heterojunctions. As displayed in the inset of Figure 3d, a distinct interface between the W_2_C and WC phases was observed, which helps regulate the electron distribution, thereby enhancing catalytic activity. In addition, a thin carbon layer was observed on the surfaces of the nanoparticles, which prevents particle aggregation and corrosion, boosts electron transfer, and provides additional catalytically active sites. Furthermore, the EDX spectrum (Figure S4b) and the EDX elemental mapping (Figure 3e and Figure S4c) showed that WC/W_2_C@NC consisted of evenly distributed C, P, N, W, and O elements on the surface of the material. The successful doping with N and P atoms, originating from the PANI and PTA precursors, respectively, contributes to the customization of the electronic structure of the catalyst, thus improving its catalytic activity.

**Figure 4:**
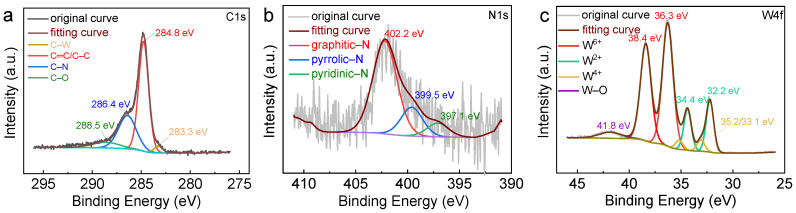
High-resolution XPS spectra of (**a**) C 1s, (**b**) N 1s, and (**c**) W 4f for WC/W_2_C@NC.

The surface composition and valence states of the catalysts play a decisive role in their activity, which was characterized using XPS analysis (Figure 4). The XPS spectra in Figure S5a illustrate the presence of N, O, C, W, and P in WC/W_2_C@NC, which is in agreement with the EDX results. The C1s spectrum (Figure 4a) was deconvoluted into four peaks, corresponding to C–W (283.3 eV), C–C/C=C (284.8 eV), C–N (286.4 eV), and C–O (288.5 eV) bonds, respectively [39]. The presence of the C–N bond confirms that nitrogen was doped into the carbon matrix after the carbonization of the PTA/PANI precursor, while the C–W bond originates from WC/W_2_C. Moreover, the presence of the oxygen-containing group (C–O) on the carbon substrate enhanced the wettability of the catalyst and substrate and facilitated its spreading in the acetonitrile phase [40]. The high-resolution N1s spectra (Figure 4b) showed three distinct peaks at 397.1, 399.5, and 402.2 eV, corresponding to pyridinic-N, pyrrolic-N, and graphite-N environments, respectively [17], further confirming N-doping. The W 4f XPS spectra (Figure 4c) were resolved into seven peaks, which could be assigned to W^2+^ (32.2/34.4 eV), W^4+^ (33.1/35.2 eV), W^6+^ (36.3/38.4 eV), and W–O bonds (41.8 eV), respectively. Among them, W^2+^ and W^4+^ originate from W_2_C and WC species, while the W^6+^ and W–O bonds result from the inevitable surface oxidation of the composites in air, as previously reported [41,42,43]. For the O1s spectra (Figure S5b), the peaks at 530.4 and 531.9 eV correspond to W–O and C–O, respectively. Finally, the high-resolution P2p spectra (Figure S5c) showed peaks at 133.2 and 134.3 eV, ascribed to the P–C and P–O bonds, respectively [44], suggesting the incorporation of P into the catalyst.

### 2.2. Evaluation of ODS Efficiencies of Catalysts

**Figure 5:**
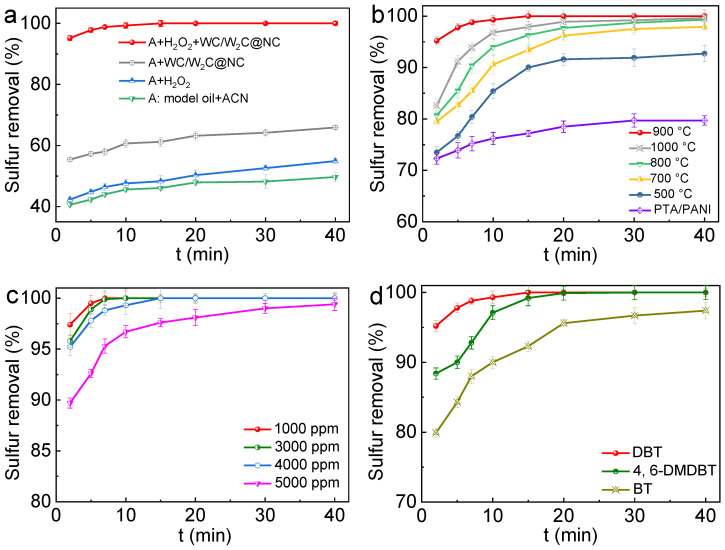
The effect on the removal of DBT by (**a**) different catalytic systems, (**b**) the derivatives obtained from calcining PTA/PANI under 500, 700, 800, 900, and 1000 °C, (**c**) different S concentrations, and (**d**) different S substrates. Reaction conditions: (**a**,**b**,**d**) 0.05 g of catalyst; n (H_2_O_2_):n (S) = 2:1; T = 60 °C; and initial sulfur content = 4000 ppm and (**c**) 0.05 g of catalyst; n (H_2_O_2_):n (S) = 2:1; and T = 60 °C.

The ODS efficiency of the model fuel oil in different catalytic systems is illustrated in Figure 5a, which demonstrates that both a catalyst and an oxidizing agent are essential for the ODS reaction. Without either a catalyst or an oxidant, sulfur removal is mainly based on the extraction effect of acetonitrile, resulting in a desulfurization rate of 49.7% after 40 min. Introducing H_2_O_2_ as an oxidant without a catalyst only slightly increases the desulfurization efficiency to 54.9%, underlining the limited oxidation capacity of H_2_O_2_ for DBT without catalytic support. Moreover, employing WC/W₂C@NC alone, in the absence of H_2_O_2_, raises the desulfurization rate to 65.9%, which is due to the strong adsorption of DBT by the porous structure of WC/W₂C@NC (Figure S6). Remarkably, combining WC/W₂C@NC with H_2_O_2_ enables the complete removal of DBT from the simulated oil within only 15 min, proving its superior catalytic oxidation efficiency. Furthermore, the HPLC analysis shown in Figure S7 confirms the sequential oxidation of DBT to dibenzothiophene sulfoxide (DBTO) and, subsequently, to dibenzothiophene sulfone (DBTO_2_), with DBTO_2_ identified as the sole product in the acetonitrile phase. These results provide conclusive evidence that DBT is completely oxidized to DBTO_2_ by H_2_O_2_ in the presence of WC/W₂C@NC catalysis. The exceptional catalytic oxidation activity of WC/W_2_C@NC, surpassing that of most tungsten-based catalysts reported in the literature (Table 1), underscores its significant potential for application in the deep desulfurization of fuel oil.

Moreover, the annealing temperature plays a crucial role in determining the structure of the calcined products, thereby impacting their ODS performance. As evident from Figure 5b, the PTA/PANI precursor exhibited a certain level of ODS activity, achieving a DBT removal rate of 79.7% within 40 min. This effect is primarily attributed to the catalytic oxidation of H_2_O_2_ by the PTA active sites. However, the removal process does not continue beyond this point, resulting in its incomplete desulfurization. In addition, the catalysts prepared at different annealing temperatures displayed varied activities, with both excessively high and low temperatures adversely affecting the ODS efficiency. Notably, among all the catalysts, WC/W_2_C@NC (calcined at 900 °C) exhibited the most outstanding ODS performance, reducing the DBT content by 99.3% within 10 min and achieving complete removal within 15 min. The ODS activity of WC/W_2_C@NC even surpassed that of several W-based catalysts reported in the literature, as shown in Table 1. The XRD analysis (Figure 2b) indicated that the active phase formed at 900 °C is a WC/W_2_C heterojunction, which is known for its excellent catalytic centers compared to other phases such as PTA (500 °C), WO_3_ (700 °C), and W/W_2_N (800 °C). The WC and W_2_C phases created a rich heterointerface that facilitated efficient electron transfer between the two phases, thereby rationally tuning the electronic structure of the active sites and significantly enhancing the intrinsic catalytic activity. In addition, the uniform distribution of WC/W_2_C heterojunctions within the porous catalyst significantly increased the exposure of the active sites, ultimately conferring exceptional catalytic performance to the WC/W_2_C@NC catalyst.

Furthermore, the adaptability of the developed WC/W_2_C@NC catalyst was evaluated for fuel oils with varying S concentrations (ranging from 1000 to 5000 ppm) and diverse S-containing compounds (including BT and 4, 6-DMDBT, each with a S content equal to 4000 ppm). As depicted in Figure 5c, the WC/W_2_C@NC catalyst effectively removed DBT at sulfur concentrations of 1000, 3000, and 4000 ppm within 7, 10, and 15 min, respectively. A removal rate of 99.4% was achieved within 40 min, even at the highest sulfur concentration of 5000 ppm, indicating that the WC/W_2_C@NC catalyst is an effective catalyst for ODS reactions in fuel oils across a wide range of S concentrations. Subsequently, the optimal ODS reaction conditions for the WC/W_2_C@NC catalyst were established by varying the catalyst dosage and the molar ratio of H_2_O_2_ to S (n (H_2_O_2_)/n (S)). As illustrated in Figure S8 and the related discussion, the catalyst exhibited optimal catalytic activity when the catalyst dosage was 0.05 g, with a H_2_O_2_/S molar ratio of 2 mol/mol (equal to the theoretical value). Moreover, Figure 5d further demonstrates the ODS performance of the WC/W_2_C@NC catalyst for various thiophene compounds in fuel oil. The desulfurization efficiency of the catalyst followed the order DBT > 4, 6-DMDBT > BT. This trend is attributed to the electrophilic attack of reactive oxygen on S atoms, which is closely related to the electron density of the S atoms within the thiophene compounds and fundamentally drives their oxidation. Of these, BT exhibited the strongest oxidative resistance due to it having the lowest electron density (5.739). Although 4, 6-DMDBT has a higher value of electron density (5.760) than DBT (5.758), it is less easily oxidized due to the steric hindrance imposed by its methyl groups [18,35]. Nevertheless, 4, 6-DMDBT and BT still achieved 100% and 97.4% desulfurization within 30 and 40 min, respectively. These results demonstrate that the WC/W_2_C@NC catalyst has strong adaptability for ODS in fuels containing various sulfur compounds.

**Figure 6:**
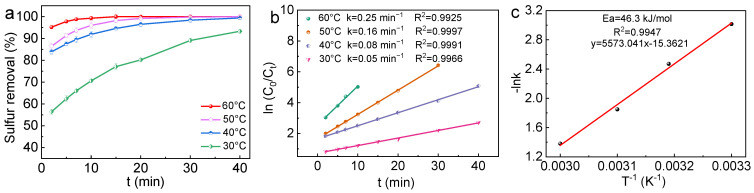
(**a**) The effect of the reaction temperature on the conversion efficiencies of DBT; (**b**) corresponding pseudo-first-order kinetic model; (**c**) Arrhenius plot for the ODS of DBT over WC/W_2_C@NC. Reaction conditions: 0.05 g of catalyst; T = 60 °C; n (H_2_O_2_):n (S) = 2:1; and initial S content = 4000 ppm.

The reaction temperature plays a critical role in activating both the catalyst and the oxidant, and a moderate reaction temperature is also essential for industrial applications. Consequently, the impact of the reaction temperature on the desulfurization efficiency of the WC/W_2_C@NC catalyst was further investigated. As depicted in Figure 6, the ODS performance of the catalyst substantially improved with increasing temperature. The desulfurization efficiency for DBT reached 99.3% within 10 min and 100% within 15 min at an optimal reaction temperature of 60 °C. The DBT removal rate remained high even at lower temperatures of 50 and 40 °C, achieving 100% and 99.4% within 40 min, respectively. Notably, near room temperature (30 °C), the desulfurization efficiency of DBT in the model fuel remained as high as 93.3% within 40 min. The quasi-first-order kinetic plot derived from Figure 6a is displayed in Figure 6b, with all data points fitting the model well with a high correlation coefficient (R^2^ > 0.99) [51]. The rate constants (k) for DBT oxidation at different temperatures were 0.25, 0.16, 0.08, and 0.05 min^−1^, with the highest kinetic constant obtained at 60 °C. According to the Arrhenius plot (Figure 6c), the activation energy (*E*a) for WC/W_2_C@NC was determined to be 46.3 kJ/mol (see Supplementary Information, Section S5). The high kinetic constant (k = 0.25 min^−1^) and relatively low value of *E*a (46.3 kJ/mol) underscore the excellent ODS capability of WC/W_2_C@NC in the presence of H_2_O_2_.

### 2.3. ODS Mechanism

**Figure 7:**
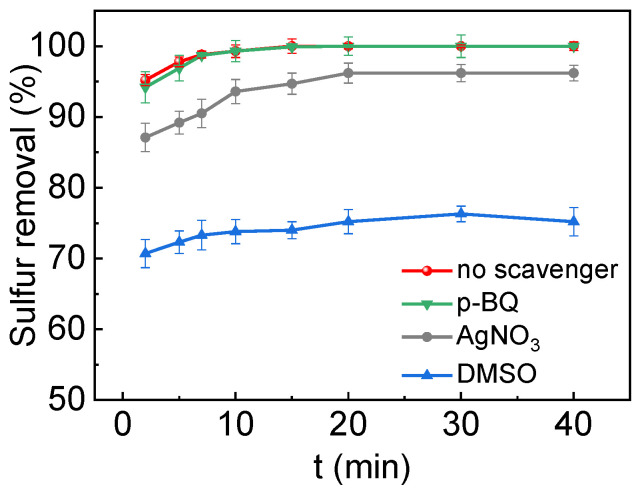
Free radical quenching test. Reaction conditions: 0.05 g of catalyst; T = 60 °C; n (H_2_O_2_):n (S) = 2:1; and initial S content = 4000 ppm.

In order to deeply elucidate the ODS mechanism of WC/W_2_C@NC, a series of experiments were conducted using p-benzoquinone (p-BQ), dimethyl sulfoxide (DMSO), and silver nitrate (AgNO_3_) as scavengers for superoxide anion radicals (O_2_•^−^), hydroxyl radicals (HO•), and electrons (e^−^), respectively [52]. As depicted in Figure 7, introducing BQ did not notably impact the desulfurization performance, indicating that the ODS reaction does not involve O_2_•^−^. In contrast, adding DMSO significantly affected the desulfurization rate, which decreased from 100% to 74.0% after 15 min. This finding strongly supports the critical role of HO• in the catalytic ODS process. Additionally, the inclusion of AgNO_3_ also affected ODS performance, reducing the desulfurization rate to 94.7% after 15 min. It can be inferred that during the oxidation of DBT, the ODS reaction primarily involves the participation of HO• radicals and e^−^, rather than O_2_•^−^ radicals. Further confirmation of the presence of HO• radicals was obtained through electron paramagnetic resonance (EPR) experiments (Figure S9). Using 5, 5-Dimethyl-1-pyrroline-N-oxide (DMPO) as a radical scavenger, typical peak signals of DMPO-HO• were found in the EPR spectrum, with a quadruple peak area ratio of 1:2:2:1 in the WC/W_2_C@NC system, consistent with previous reports [39]. This result validates the formation of HO• within the system. Moreover, the nitrogen-doped carbon matrix derived from PANI effectively prevents the aggregation of WC/W_2_C nanoparticles. The high-spin and charge-dense nitrogen atoms in the carbon matrix enhance the electronic loading capacity and facilitate efficient electron transfer to the closely bound WC/W_2_C nanoparticles, thereby promoting the decomposition of H_2_O_2_ into HO• radicals [53].

**Figure 8:**
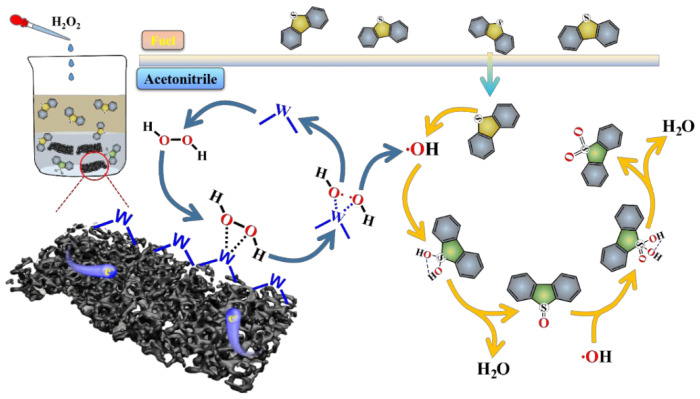
ODS mechanism of the model oil over WC/W_2_C@NC.

A plausible mechanism for the ODS process is illustrated in Figure 8, based on the experimental findings discussed above. Initially, sulfur-containing compounds in the n-octane phase are efficiently transferred into the acetonitrile phase. Concurrently, H_2_O_2_ is chemically adsorbed onto the W sites of the catalyst through O–O bond coordination. This adsorption facilitates the subsequent decomposition of H_2_O_2_ into HO• radicals through the synergistic effects of WC and the W_2_C heterojunction [17]. Notably, the electron transfer from the NC layer to the W sites enhances the electron density on the W atoms, significantly promoting the cleavage of the O–O bond and thereby accelerating the generation of HO• radicals [35,54]. Following this, the adsorbed sulfur-containing compounds on the catalyst surface undergo oxidation to dibenzothiophene oxide (DBTO), which is then completely oxidized to dibenzothiophene dioxide (DBTO_2_) by the HO• radicals. The ODS process for the model oil is demonstrated by HPLC (see Figure S7), during which DBT in the acetonitrile phase continuously oxidizes to DBTO_2_, while the remaining DBT in the n-octane phase is sequentially extracted into the acetonitrile phase for further oxidation until all the DBT is transformed and fully converted to DBTO_2_.

### 2.4. Recyclability and Reusability of Catalyst

**Figure 9:**
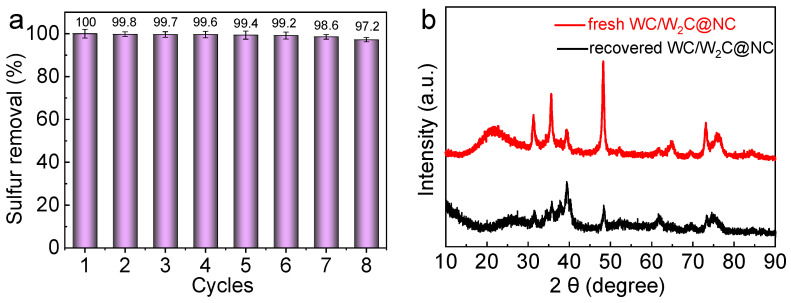
(**a**) Recycling performance of WC/W_2_C@NC for DBT removal; (**b**) XRD spectra of WC/W_2_C@NC before and after ODS reaction. Reaction conditions: (**a**) 0.05 g of catalyst; T = 60 °C; n (H_2_O_2_):n (S) = 2:1; and initial S content = 4000 ppm.

To comprehensively evaluate the stability of the WC/W_2_C@NC catalyst, recycling performance tests were further conducted. After each ODS reaction cycle, the acetonitrile phase containing the catalyst was effectively separated from the upper oil phase using high-speed centrifugation. Subsequently, the catalyst phase was thoroughly washed multiple times with ethanol and acetonitrile and then dried in an oven at 60 °C for reuse in subsequent cycles. As shown in Figure 9a, the WC/W_2_C@NC catalyst maintained stable ODS performance after eight cycles, achieving an efficiency of 97.2% within 15 min, with only a negligible decrease, potentially due to minor mass loss during the recovery process. SEM images of the recycled catalyst (Figure S10) reveal that the WC/W_2_C@NC catalyst retained its unique three-dimensional porous structure even after multiple cycles. No significant structural collapse or particle agglomeration was observed, and the structure remained consistent with that of the freshly prepared catalyst, demonstrating the catalyst’s structural stability. Additionally, XPS analysis (Figure S11) showed that the chemical states and forms of elements such as C, N, W, O, and P in both the fresh and recycled WC/W₂C@NC catalysts were nearly identical. This was further corroborated by EDX analysis (Figure S12), confirming that the catalyst maintained excellent stability even after multiple recycling cycles. Finally, the XRD patterns of the recycled catalyst exhibited the same characteristic peaks as those of the freshly prepared catalyst (Figure 9b), indicating that its crystal structure remained largely unchanged after multiple uses, further underscoring its stability and reusability.

## 3. Materials and Methods

### 3.1. Materials

The drugs and reagents used in this study were obtained from Shanghai Aladdin, McLean Co., Ltd., Shanghai, China, and other suppliers without any further purification or modification before use. More details are provided in Section S1 of the Supplementary Information.

### 3.2. Characterization

The prepared catalysts were investigated using various characterization tools, including X-ray diffraction (XRD), scanning electron microscopy (SEM), X-ray photoelectron spectroscopy (XPS), transmission electron microscopy (TEM), electron paramagnetic resonance (EPR), and thermogravimetry (TG). Further information can be found in Section S2 of the Supplementary Information.

### 3.3. Evaluation of the Catalytic Activity

A model fuel with 4000 ppm of sulfur was prepared by dissolving specific amounts of sulfur compounds (DBT, BT, and 4, 6-DMDBT) in n-octane. In a typical ODS process, desulfurization studies were carried out in a three-neck round-bottomed flask equipped with a glass condenser cooled by circulating cold water to prevent solvent evaporation. To establish extraction equilibrium, 10 mL of acetonitrile (ACN) was added to 20 mL of model oil, and the mixture was stirred for 30 min to form a biphasic system. Subsequently, 10 mL of an ACN solution containing the catalyst and 30 wt% H_2_O_2_ was introduced at 60 °C to initiate the ODS reaction. During the reaction, 50 µL samples were periodically extracted from both the upper n-octane phase and the lower acetonitrile phase and then diluted with 3 mL of n-octane and acetonitrile, respectively. The sulfur content was monitored utilizing high-performance liquid chromatography (HPLC) with an external standard technique (refer to Supplementary Information of Part 4 for detailed detection conditions). The desulfurization rate (*y*) was determined using Equation (1).

(1) $$y=\frac{C_{0}-C_{t}}{C_{0}}\times 100\%$$

## 4. Conclusions

In summary, a three-dimensional porous WC/W_2_C@NC heterojunction catalyst was successfully prepared via in situ pyrolysis carbonization, using PTA/PANI as a precursor, and subsequently applied in the ODS of fuels. During the pyrolysis process, the derived carbon carriers effectively inhibited the excessive agglomeration of WC/W_2_C nanoparticles. Consequently, the optimized WC/W_2_C@NC catalyst demonstrated excellent ODS performance and reusability in a biphasic system, achieving 100% removal of 4000 ppm of DBT within 15 min at 60 °C in the presence of 0.03 g of WC/W_2_C@NC and at a H_2_O_2_/S molar ratio of 2, as well as retaining activity over eight reuse cycles with negligible loss. The exceptional ODS activity of WC/W_2_C@NC can be attributed to the strong electron-donating nature of the NC layer, the synergistic effect of WC and W_2_C active species, and the presence of WC/W_2_C heterojunctions. The WC/W_2_C heterojunctions facilitate electron transfer, while the electron-donating effect of the NC layer enables effective electron accumulation at W sites, promoting O–O bond cleavage to generate HO• radicals. Additionally, the efficient electron transfer between the NC layer and WC/W_2_C heterojunctions stimulated a strong electrostatic interaction between the active components and the support, thereby improving the stability of the catalyst. This study introduces a versatile strategy for in situ transformation, enabling the tailoring of the catalyst surface structure to optimize catalytic performance.

## Figures and Tables

**Table 1:** Comparison of oxidation of DBT by different catalysts.

Catalysts	n (H_2_O_2_): n (S)	Dosage (g)	T (°C)	Initial S Conc. (ppm)	Time (min)	Removal Efficiency (%)	Reaction Rate Constant (k (min^−1^))	Refs
C_16_-PW_9_/SiO_2_	3	0.01	50	500	100	100	0.24	[45]
flower-like WO_3_·H_2_O	8	0.13	70	4000	60	98.7	0.05	[46]
[MIMPs]_3_PMo_6_W_6_O_40_	2.5	0.05	60	500	40	100		[47]
CNT@C_4_VW_12_	6	0.01	60	500	30	100	-	[48]
W_2_C@C	3	0.04	50	500	40	100	0.146	[24]
MoO_3_/NGO	4	0.05	60	500	160	95	-	[49]
PTA@MIL-101(Cr)	5	0.05	60	500	120	84.1	-	[50]
VO-MoO_2_@NC	4	0.08	70	500	40	100		
WC/W_2_C@NC	2	0.05	60	4000	15	100	0.24	This work
	2	0.05	60	3000	10	100		
	2	0.05	60	1000	7	100		
	2	0.05	60	5000	40	99.4		

## Data Availability

Data are contained within the article and Supplementary Materials; further inquiries can be directed to the corresponding author.

## Supplementary Materials

The following Supporting Information can be downloaded at https://www.mdpi.com/article/10.3390/molecules30030617/s1: Figure S1: UV-Vis spectra of PTA/PANI composite and its annealed products at different temperatures; Figure S2: TG of spectra of PTA/PANI under N_2_ atmosphere with a heating rate of 10 °C/min; Figure S3: SEM of PTA/PANI at different magnifications; Figure S4: The corresponding elemental mapping images of O for WC/W_2_C@NC; Figure S5: (a) XPS survey spectrum: high-resolution spectra of (b) O 1s and (c) P 2p for WC/W_2_C@NC; Figure S6: Model oils containing DBT + ACN system added with WC/W_2_C@NC; Figure S7: HPLC chromatograms of (a) n-octane phases and (b) acetonitrile phases (catalyst: 0.05 g; n (H_2_O_2_):n (S) = 2:1; T = 60 °C; initial sulfur content = 4000 ppm); Figure S8: The effect of (a) catalyst dosage and (b) n (H_2_O_2_)/n (S) on ODS activity; Figure S9: EPR spectra of the ODS reaction system. Figure S10: SEM image of recovered WC/W_2_C@NC. Figure S11: XPS spectra of (a) full survey; (b) C1s; (c) N1s; (d) W4f; (e) O1s; and (f) P2p for fresh and recovered WC/W_2_C@NC. Figure S12: EDX spectrum of recovered WC/W_2_C@NC.
