# Supplementary material for: In Situ Formation of WC/W2C Heterostructures on N-Doped Carbon for Deep Oxidative Desulfurization of Fuel Oil
Source: Molecules. 2025 Jan 31;30(3):617. doi: 10.3390/molecules30030617 (PMC11821058; doi:10.3390/molecules30030617)
Supplement: Supplementary file 1 [file molecules-30-00617-s001.zip › molecules-3418555-supplementary.pdf]

In situ formation of WC/W_2C Heterostructures on N-doped carbon for deep oxidative desulfurization of fuel oil

Peng Zuo, Fuyan Zhao, Fanfan Liu, Jinpei Hei, Guozheng Lv, Xianzong Huang, Jun Zhang, Meng Zhang, Yefeng Liu * and Tao Ma *

Engineering Technology Research Center of Preparation and Application of Industrial Ceramics of Anhui Province, Engineering Research Center of High-frequency Soft Magnetic Materials and Ceramic Powder
Materials of Anhui Province, School of Chemistry and Material Engineering, Chaohu University,
Chaohu 238000, China; zp@chu.edu.cn (P.Z.); fyzhao@chu.edu.cn (F.Z.); lff@chu.edu.cn (F.L.); 053074@chu.edu.cn (J.H.);
14755796996@163.com (G.L.); 15156256409@163.com (X.H.);
13339143725@163.com (J.Z.); 15357423324@163.com (M.Z.)
* Correspondence: lyf@chu.edu.cn (Y.L.); matao@chu.edu.cn (T.M.)

S1. Chemicals and reagents

Dibenzothiophene (DBT), benzothiophene (BT), and 4, 6-dimethyldibenzothiophene (4, 6-DMDBT) were purchased from Shanghai Aladin Biochemical Technology Co., LTD. Aniline, ammonium persulfate (APS), and $H_3PW_{12}O_{40}\cdot xH_2O$ (phosphotungstic acid, PTA) were all from Shanghai Maclin Biochemical Co., LTD. Anhydrous methanol, acetonitrile, n-octane, and hydrogen peroxide (H_2O_2, 30 wt.%) were purchased from Tianjin Damao Chemical Reagent Factory.

S2. Characterization apparatus

The apparatus used included a Fourier Infrared Spectrometer (FT-IR, KBr method, L1600300 Spectrum Two Lita Model, Liantrisant, UK); Double-Beam UV–Visible Spectrophotometer (UV-Vis, T9CS, PUXI, China); X-ray diffractometer (XRD, Bruker D8 Advance, Bruker Corporation, Germany); thermogravimetric instrument (TG, METTLER TOLEDO TGA/DSC 3+, Switzerland); Scanning electron microscope (SEM, JIEKE TESCAN MIRA LMS, Czech Republic); X-ray photoelectron spectroscopy instrument (XPS, Thermo Scientific ESCALAB 250Xi, USA); Transmission Electron Microscope (TEM, FEI Talos 200S, American); High-Resolution Transmission Electron Microscope (HRTEM, FEI Talos 200S, American); High-Performance Liquid Chromatograph (HPLC, L-3000, Beijing Puyuan fine Electronics Technology Co., Ltd, Beijing, China); and EPR instrument (MiniSpcope MS 5000, Magnettech, Germany) with 5, 5-dimethyl-1-pyrrolidine-N-oxide (DMPO) as an electron-capturing reagent.

S3. Synthesis of PTA/PANI

The PTA/PANI precursor was prepared by a simple one-step ice-bath method. First, 0.6 mL of aniline was

dissolved in 20 mL of hydrochloric acid solution (2 mol·L^{-1}), and 0.6 g of PTA dissolved in 40 mL of the solution was added drop by drop under stirring. Subsequently, 1 g of ammonium persulfate (APS) was weighed, dissolved in 8 mL of deionized water, and added to the above solution; it was again stirred under ice-bath conditions for 24 h, centrifuged, washed with deionized water and ethanol many times, and dried. The dark green loaded precursor PTA/PANI was obtained.

S4. HPLC test conditions

The HPLC test conditions were as follows: A C18 reversed-phase column (200 mm×4.6 mm, 5 μm ID) was used. The column temperature was maintained at 35 °C. The detection wavelengths were specifically set to 254 nm for DBT, BT, and 4, 6-DMDBT, respectively. The mobile phase comprised a mixture of methanol and water in a volume ratio of 9:1, which flowed through the column at a rate of 1.0 mL·min^{-1}.

S5. Kinetic studies of ODS and the apparent activation energy

The k value in Figure 6b can be obtained from the following formula:

$$\ln\frac{C_0}{C_t} = kt \quad \text{(S1)}$$

where k is the rate constant, C_0 is the initial S content of the model oil, and C_t is the S content at time t.

A plot of ln k against 1/T is shown in Figure 6c according to the Arrhenius equation (S2):

$$\ln k = -\frac{E_a}{RT} + \ln A \quad \text{(S2)}$$

where E_a is the apparent activation energy, kJ/mol; R is the molar gas constant, J/(mol·K); T is the thermodynamic temperature, K; A is the pre-exponential factor; and k is the reaction rate constant, min^{-1}. According to the linear relationship between -ln k and $1/T$ in Figure 6c, E_a is estimated as 46.3 kJ·mol^{-1}.

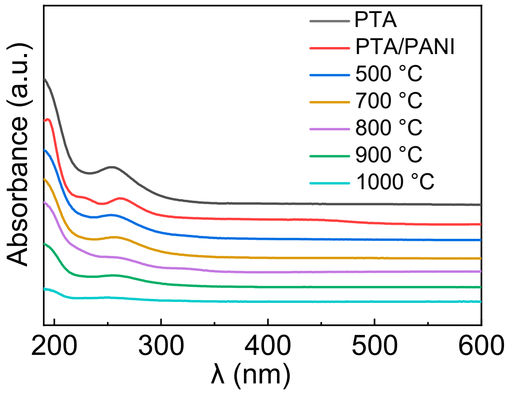

Figure S1. UV-Vis spectra of PTA/PANI composite and its annealed products at different temperatures.

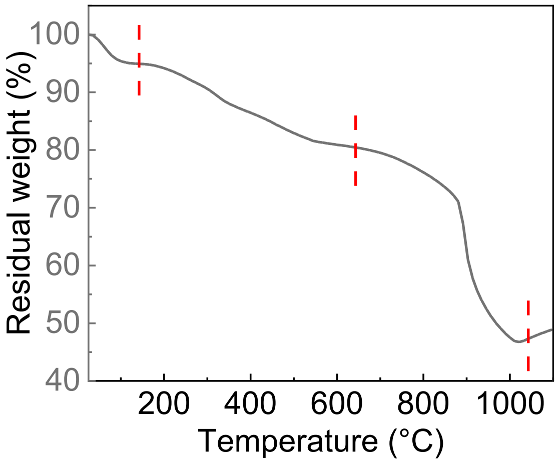

Figure S2. TG of spectra of PTA/PANI under N_2 atmosphere with a heating rate of 10 ℃ / min.

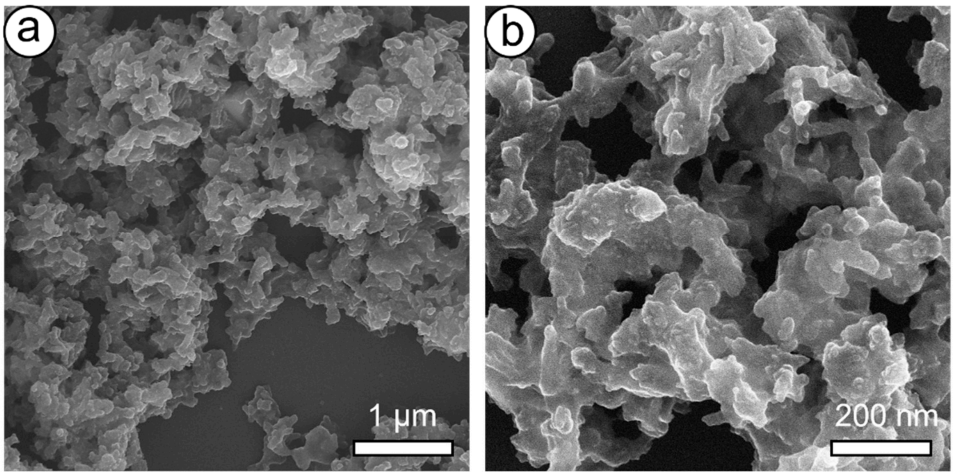

Figure S3. SEM of PTA/PANI at different magnifications.

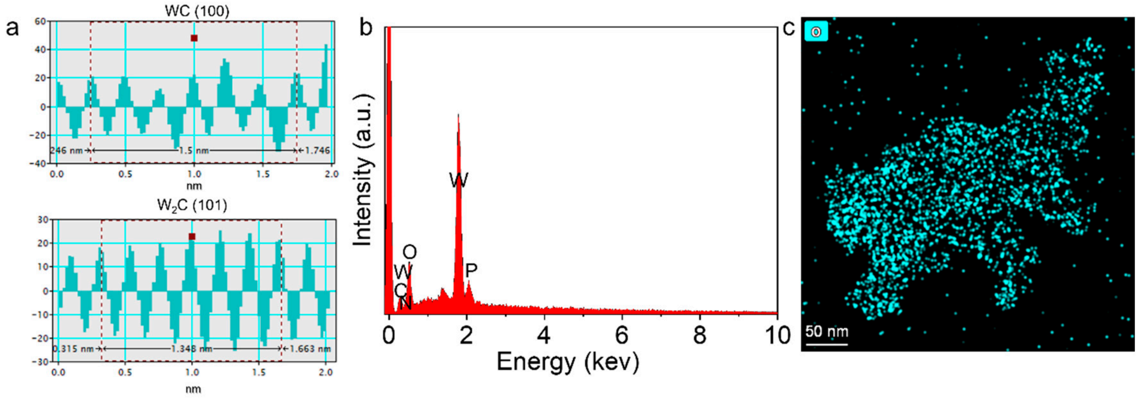

Figure S4. (a) Average of six measured lattice spacings, (b) EDX spectrum of WC/W_2C@NC, and (c) corresponding elemental mapping images of O for WC/W_2C@NC.

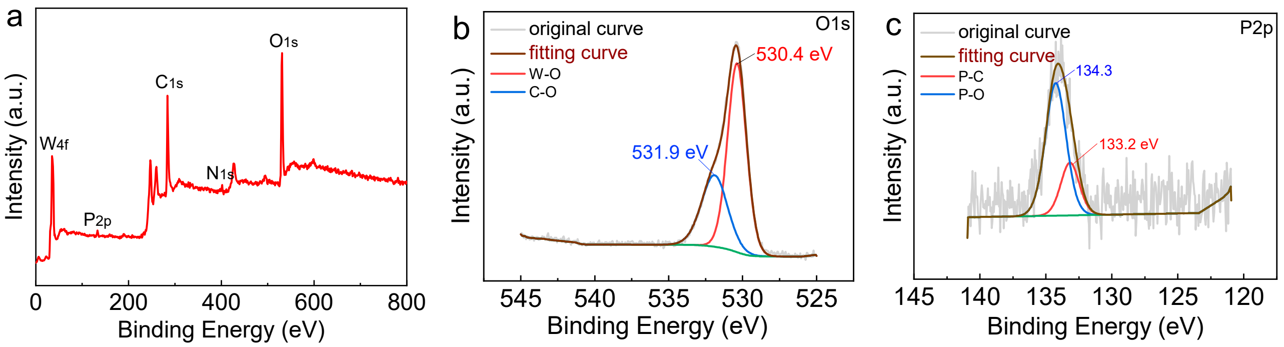

Figure S5. (a) XPS survey spectrum: high-resolution spectra of (b) O 1s and (c) P 2p for WC/W2C@NC.

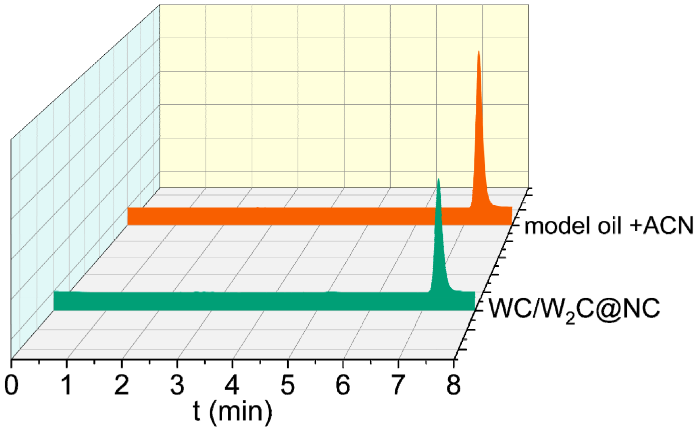

Figure S6. Model oils containing DBT+ACN system added with WC/W2C@NC.

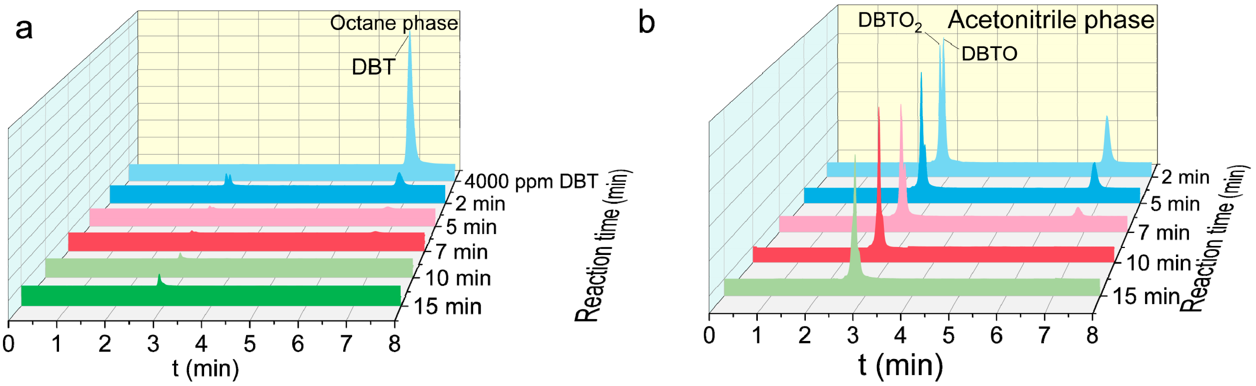

Figure S7. HPLC chromatograms of (a) n-octane phases and (b) acetonitrile phases (catalyst: 0.05 g; n(H_2O_2):n(S)=2:1; T = 60 °C; initial sulfur content = 4000 ppm).

It is universally acknowledged that the dosage of catalyst and the H_2O_2/S molar ratio are pivotal parameters in optimizing the performance of the oxidative desulfurization (ODS) reaction. To ascertain the optimal desulfurization process conditions, an in-depth investigation into the effects of catalyst loading and the H_2O_2/S molar ratio was conducted. As depicted in Figure S8a, a notable enhancement in sulfur removal efficiency is

observed as the catalyst dosage increases from 0.01 to 0.05 g. This enhancement is primarily attributed to the proliferation of active sites on the catalyst, leading to a reduction in the complete desulfurization time to merely 15 minutes. Nevertheless, when the catalyst dosage is further increased to 0.07 g, a decline in desulfurization efficiency is noted, likely due to the excessive catalyst-promoting ineffective decomposition of H_2O_2. Consequently, the optimal dosage of the WC/W_2C@NC catalyst is determined to be 0.05 g. On the other hand, H_2O_2, being an efficient and environmentally benign oxidant in the ODS reaction, plays a crucial role in determining the reaction outcome. The impact of the H_2O_2/S molar ratio on desulfurization efficiency is elucidated in Figure S8b. As the H_2O_2/S molar ratio increases, a gradual ascent in desulfurization efficiency is observed. Specifically, when the molar ratio reaches 2 (i.e., n(H_2O_2)/n(S)=2), a remarkable achievement of 100 % sulfur removal within 15 minutes is attained. However, a further increase in the H_2O_2 dosage results in a decrease in desulfurization efficiency. This phenomenon is primarily attributed to the introduction of excessive H_2O_2, which elevates the water content in the acetonitrile phase, consequently enhancing interfacial mass transfer resistance and decelerating mass transfer rates. These factors, in turn, impede the oxidation process of dibenzothiophene (DBT). Therefore, the optimal H_2O_2/S molar ratio is established to be 2:1.

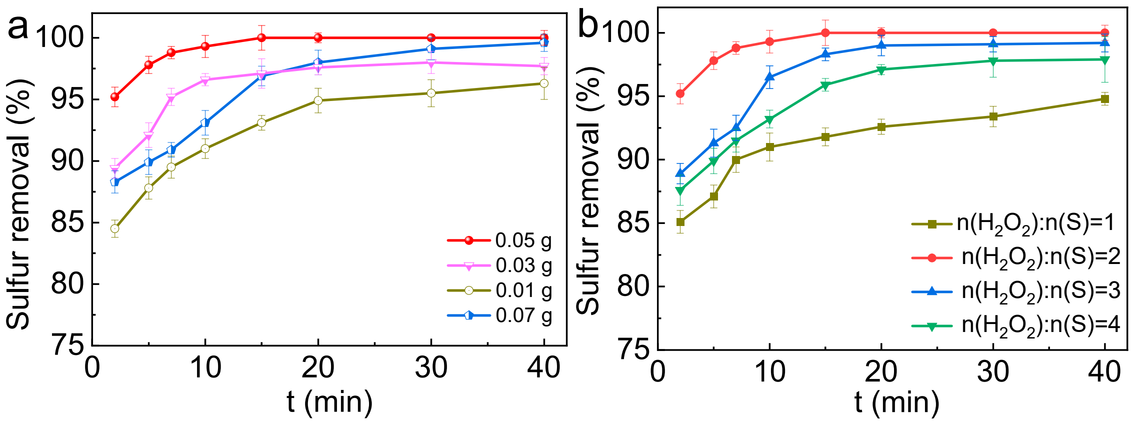

Figure S8. The effect of (a) catalyst dosage and (b) n(H_2O_2)/n(S) on ODS activity.

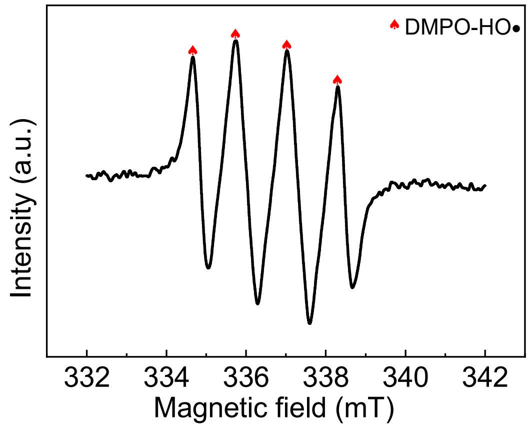

Figure S9. EPR spectra of the ODS reaction system.

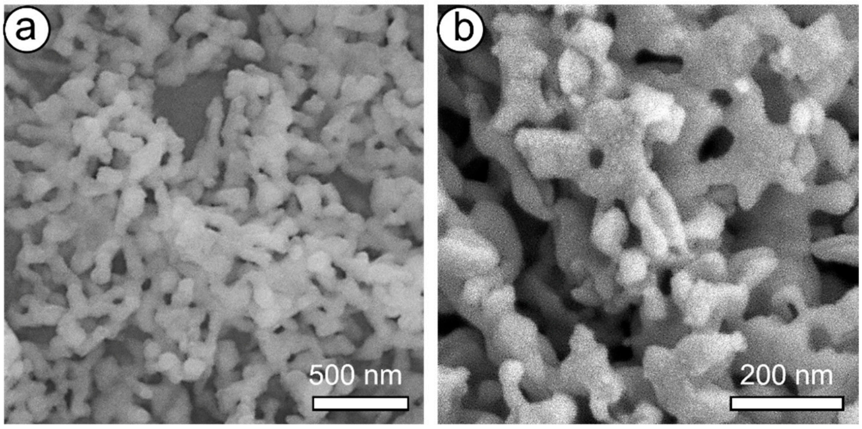

Figure S10. SEM image of recovered WC/W_2C@NC.

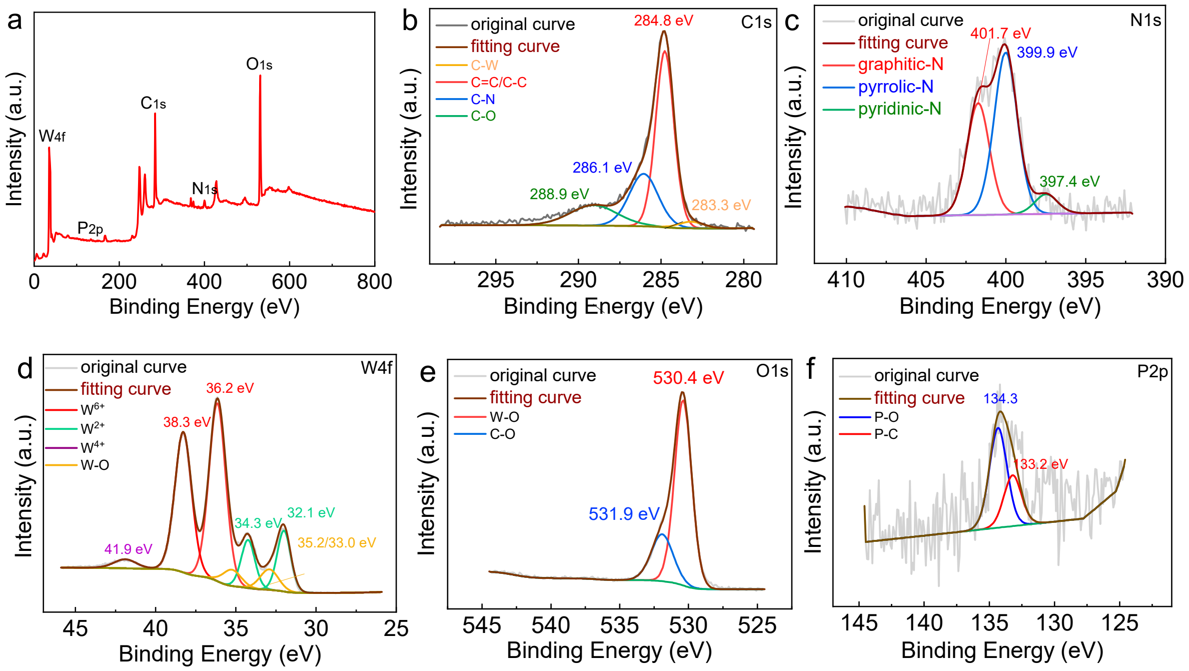

Figure S11. XPS spectra of (a) full survey; (b) C1s; (c) N1s; (d) W4f; (e) O1s; and (f) P2p for fresh and recovered

WC/W_2C@NC.

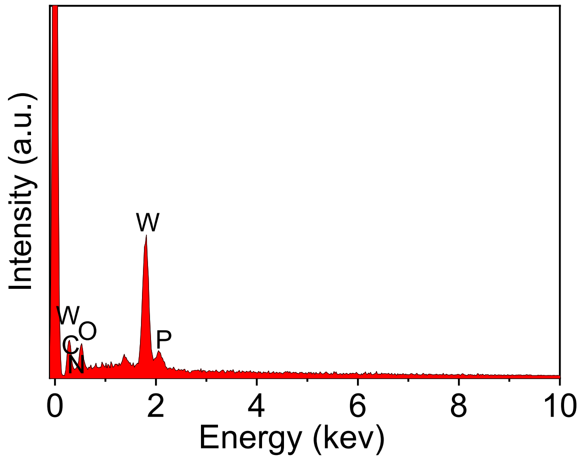

Figure S12 EDX spectrum of recovered WC/W_2C@NC.
